# Poster Session II - A234 FROM PLATE TO GUT: AGE-DEPENDENT EFFECTS OF DIETARY LIPIDS AND GUT MICROBIOTA ON CROHN’S DISEASE RISK

**DOI:** 10.1093/jcag/gwaf042.234

**Published:** 2026-02-13

**Authors:** F A Guevara Agudelo, S Jeong, R Khorasaniha, Q Li, A Waslyk, A Griffiths, H Steinhart, R Panaccione, H Armstrong, S Lee, K Croitoru, W Turpin

**Affiliations:** University of Toronto Temerty Faculty of Medicine, Toronto, ON, Canada; University of Toronto Temerty Faculty of Medicine, Toronto, ON, Canada; Lunenfeld-Tanenbaum Research Institute, Sinai Health, Toronto, ON, Canada; Lunenfeld-Tanenbaum Research Institute, Toronto, ON, Canada; Lunenfeld-Tanenbaum Research Institute, Toronto, ON, Canada; The Hospital for Sick Children, Toronto, ON, Canada; Lunenfeld-Tanenbaum Research Institute, Sinai Health, Toronto, ON, Canada; University of Calgary, Calgary, AB, Canada; University of Alberta, Edmonton, AB, Canada; Lunenfeld-Tanenbaum Research Institute, Sinai Health, Toronto, ON, Canada; University of Toronto Temerty Faculty of Medicine, Toronto, ON, Canada; Lunenfeld-Tanenbaum Research Institute, Toronto, ON, Canada

## Abstract

**Background:**

Previous studies suggest dietary lipids may influence Crohn’s disease (CD) risk. Our group previously identified gut microbial signatures predictive of CD onset. However, how dietary lipid–microbiota interactions may contribute to CD risk remains poorly understood, especially across age groups from childhood to early adulthood.

**Aims:**

To investigate age-dependent associations between dietary lipids and CD risk.

**Methods:**

We analyzed 2,342 first-degree relatives of CD patients from CCC-GEM (66 pre-CD, 2,276 controls). Participants completed food frequency questionnaires and provided stool samples at recruitment. Clustering analysis of 53 dietary lipids yielded 26 representative lipids. Stool microbiome was profiled by 16S rRNA sequencing; fecal calprotectin (FCP) measured by ELISA. Cox models assessed time-to-CD onset; age-stratified regression examined lipid-FCP associations across pediatric (<15), young adult (15-25), and adult (25-40) groups.

**Results:**

We observed age-dependent associations between dietary lipids, inflammation, and CD risk. In the overall cohort, alpha-linolenic acid (ALA) (β = 177.15, p = 0.007) and behenic acid (β = 159.70, p = 0.005) associated with elevated FCP. Age-stratified analysis revealed these pro-inflammatory effects were pediatric-specific: ALA (β = 0.25, p = 0.035), behenic acid (β = 0.36, p = 0.025), and trans-palmitoleic acid (β = 0.98, p = 0.046) elevated FCP in children but not in young adults or older adults (all p > 0.05). Despite elevating inflammation in children, protective CD associations emerged exclusively in young adults (15-25): ALA (HR = 0.53, p < 0.001), behenic acid (HR = 0.75, p = 0.042), and tetracosenoic acid (HR = 0.77, p < 0.001), with no effects in pediatric or older adult groups. These associations remained significant after FCP adjustment (ALA HR = 0.61, p = 0.008; behenic acid HR = 0.72, p = 0.020; tetracosenoic acid HR = 0.81, p = 0.043). In the overall cohort, cholesterol associated with increased CD risk (HR = 1.37, p = 0.04). Trans-palmitoleic acid correlated with *R. torques* only in adults (ρ = 0.07-0.09, p < 0.01).

**Conclusions:**

Our findings reveal age-specific lipid associations with CD risk, with inverse associations in young adults (15-25) but not pediatric (<15) or older adults (25-40). While ALA and behenic acid were associated with reduced CD risk in young adults independent of FCP, their pro-inflammatory effects in children without corresponding risk reduction suggest age-dependent mechanisms requiring validation in prospective cohorts.

**Funding Agencies:**

CCC, CIHRHelmsley Charitable Trust, and International Organization for the Study of Inflammatory Bowel Diseases (IOIBD)

